# Oxidative modification of albumin in the parenchymal lung tissue of current smokers with chronic obstructive pulmonary disease

**DOI:** 10.1186/1465-9921-11-180

**Published:** 2010-12-22

**Authors:** Tillie L Hackett, Marco Scarci, Lu Zheng, Wan Tan, Tom Treasure, Jane A Warner

**Affiliations:** 1 School of Medicine, University of Southampton, Southampton, UK; 2 James Hogg Research Centre, Heart + Lung Institute, University of British Columbia, Vancouver, Canada; 3 Department of Thoracic Surgery, Guy's Hospital, Great maze pond, London, UK

## Abstract

**Background:**

There is accumulating evidence that oxidative stress plays an important role in the pathophysiology of chronic obstructive pulmonary disease (COPD). One current hypothesis is that the increased oxidant burden in these patients is not adequately counterbalanced by the lung antioxidant systems.

**Objective:**

To determine the levels of oxidised human serum albumin (HSA) in COPD lung explants and the effect of oxidation on HSA degradation using an *ex vivo *lung explant model.

**Methods:**

Parenchymal lung tissue was obtained from 38 patients (15F/23M) undergoing lung resection and stratified by smoking history and disease using the GOLD guidelines and the lower limit of normal for FEV_1_/FVC ratio. Lung tissue was homogenised and analysed by ELISA for total levels of HSA and carbonylated HSA. To determine oxidised HSA degradation lung tissue explants were incubated with either 200 μg/ml HSA or oxidised HSA and supernatants collected at 1, 2, 4, 6, and 24 h and analysed for HSA using ELISA and immunoblot.

**Results:**

When stratified by disease, lung tissue from GOLD II (median = 38.2 μg/ml) and GOLD I (median = 48.4 μg/ml) patients had lower levels of HSA compared to patients with normal lung function (median = 71.9 μg/ml, P < 0.05). In addition the number of carbonyl residues, which is a measure of oxidation was elevated in GOLD I and II tissue compared to individuals with normal lung function (P < 0.05). When analysing smoking status current smokers had lower levels of HSA (median = 43.3 μg/ml, P < 0.05) compared to ex smokers (median = 71.9 μg/ml) and non-smokers (median = 71.2 μg/ml) and significantly greater number of carbonyl residues per HSA molecule (P < 0.05). When incubated with either HSA or oxidised HSA lung tissue explants rapidly degraded the oxidised HSA but not unmodified HSA (P < 0.05).

**Conclusion:**

We report on a reliable methodology for measuring levels of oxidised HSA in human lung tissue and cell culture supernatant. We propose that differences in the levels of oxidised HSA within lung tissue from COPD patients and current smokers provides further evidence for an oxidant/antioxidant imbalance and has important biological implications for the disease.

## Background

There is accumulating evidence that oxidative stress plays an important role in the pathophysiology of chronic obstructive pulmonary disease (COPD) (1). In particular, studies have demonstrated elevated oxidative stress is associated with both severity of disease and episodes of exacerbation (2). The elevated oxidative stress in these patients is thought to result both directly from inhaled oxidants in cigarette smoke or pollution and indirectly due to the release of reactive oxygen species (ROS) generated by various inflammatory, immune and epithelial cells (3). One current hypothesis is that the increased oxidant burden in these patients is not adequately counterbalanced by the lung antioxidant systems, leading to enhanced pro-inflammatory gene expression and protein release, inactivation of antiproteinases, and as a consequence oxidative tissue injury.

The antioxidants present in serum, airway mucosa, alveolar lining fluid and cells include mucin, superoxide dismutase, glutathione, uric acid, ascorbic acid, and albumin. Human serum albumin (HSA) is a single non-glycosylated polypeptide containing 35 cysteine residues all involved in the formation of stabilising disulphide bonds except ^34^cysteine. In plasma, this free thiol group is quantitatively the most important scavenger of oxidants (4-6), and is thus an important antioxidant within the body(7).

The formation of carbonyl groups on amino acid residues as a result of free radical-initiated reactions is well documented as a marker of protein degradation and turnover (8, 9). In fact the oxidative modification of proteins and lipids has been implicated in the etiology of a number of diseases including atherogenesis and diabetes (10, 11). In particular oxidised HSA is a reliable marker of oxidative stress in patients with chronic renal failure and individuals on hemodialysis therapy (12). In light of these findings the quantification of carbonyl residues may provide further evidence to support a role of oxidative stress in COPD pathology. There are several methodologies for the quantification of carbonyl residues; in the majority of them 2,4-dinitrophenyl hydrazine is allowed to react with the protein carbonyls to form the corresponding hydrazone, which can be analysed optically by radioactive counting or immunohistochemistry. In this study we have adapted a previously published methodology based on ELISA to analyse the levels of carbonylated HSA in human lung tissue from COPD patients (13). In addition, we have investigated the effect of oxidation on HSA degradation within human lung tissue explants.

## Methods

### Patient characteristics for human lung tissue experiments

Parenchymal lung tissue from the normal margin surrounding the tumour site was obtained from 38 patients (15F/23M) undergoing resection for carcinoma at Guy's Hospital London. The study was approved by the St Thomas' Hospital Research Ethics committee, reference number EC01/047, and all volunteers gave their signed informed consent. The Global Initiative for Chronic Obstructive Pulmonary Disease (GOLD) guidelines were used to stratify patients with COPD by disease severity based on measurements of airflow limitation during forced expiration (14, 15). Each stage is determined by the volume of air that can be forcibly exhaled in one second (FEV_1_) and by the ratio of FEV_1 _to the forced vital capacity (FVC); lower stages indicate less severe disease. Using the GOLD guidelines our patient cohort was stratified into the following groups, GOLD I (FEV_1_/FVC < 70%, FEV_1 _≥ 80% predicted), GOLD II (FEV_1_/FVC < 70%, 50% ≤ FEV_1 _< 80% predicted) and individuals with normal lung function (FEV_1_/FVC > 70%, FEV_1 _≥ 90% predicted). Table 1 shows the number of patients in each GOLD stage and their demographics which include age, gender, lung function and smoking history. The patient cohort was also reclassified using the prediction equations from the National Health and Nutrition Examination Survey (NHANES) III (16) from the United States and the Health Survey for England (HSE) (17) to determine the lower limit of normal (LLN) for FEV_1_/FVC. This analysis was performed using SPSS 14.0 for Windows (SPSS, Chicago, Illinois, USA), data are given in Table 2. For the purposes of this study ex-smokers were defined as that had given up smoking for ≥3 years to ensure for smoking cessation. All demography data was available up to the date of surgery and none of the subjects were treated with inhaled or oral corticosteroids or bronchodilators.

**Table 1:** Patient characteristics of subjects prior to the removal of lung tissue

Classification	Normal Lung Function **FEV**_**1**_**/FVC > 70%** **FEV**_**1 **_**≥ 90%** predicted	GOLD I **FEV**_**1**_**/FVC ≤ 70%** **FEV**_**1 **_**≥ 80%** Predicted	GOLD II **FEV**_**1**_**/FVC ≤ 70%** **50% ≤ FEV**_**1 **_**< 80%** Predicted
No. subjects	16	13	9
Age	64.7 ± 14.1	68.2 ± 9.9	64.3 ± 12.3
Gender	6F	7F	2F
	10M	6M	7M
Pre- bronchodilator FEV1/FVC	0.78 ± 0.08	0.62 ± 0.04	0.53 ± 0.1
Smoking status	6 current smokers	5 current smokers	7 current smokers
	8 ex-smokers	5 ex-smokers	2 ex-smokers
	2 non-smokers	3 non-smokers	

Tissue samples were taken from 38 patients. Patient details including age, gender, lung function given as the ratio of air that can be forcibly exhaled in one second (FEV1) to the forced vital capacity (FVC) pre-bronchodilator use and smoking status. Data given are the mean ± SD of each group.

**Table 2:** Reclassification of subjects using lower limit of normal FEV1/FVC to define COPD

Classification	Normal Lung Function	GOLD I	GOLD II
No. subjects	12	8	11
Age	63.3 ± 4.7	71.4 ± 2.3	62.5 ± 10.6
Gender	4F	4F	4F
	8M	4M	7M
Height (M)	1.71 ± 0.01	1.68 ± 0.03	1.74 ± 0.1
Weight (Kg)	81.0 ± 4.4	67.29 ± 4.3	82.3 ± 9.7
LLN FEV1 predicted	0.91 ± 0.1	0.87 ± 0.02	0.65 ± 0.3
Smoking status	6 current smokers	4 current smokers	7 current smokers
	5 ex-smokers	3 ex-smokers	2 ex-smokers
	2 non-smokers	2 non-smokers	

Tissue samples were taken from 31 patients. Patient details including age, gender, height, weight, lung function given as lower limit of normal (LLN) of air that can be forcibly exhaled in one second (FEV1) and smoking status. Data given are the mean ± SD of each group.

### Preparation of human lung tissue for primary cell culture

Lung tissue was finely chopped using dissection scissors into fragments during several washes with Tyrode's buffer containing 0.1% sodium bicarbonate. 5-6 explants (total weight approx. 30 mg) were incubated in a 24 well plate with RPMI-1650 medium containing 1% penicillin, 1% streptomycin and, 1% gentamycin at 37°C in 5% carbon dioxide/air for 16 hours (18). Tissue was then either incubated with 200 μg/ml HSA or oxidised HSA and lung tissue and supernatant were harvested at 1, 2, 4, 6, and 24 hour time points, weighed and stored at -80°C.

### Human Serum Albumin ELISA

For measuring total levels of HSA in samples we developed a specific ELISA assay. Briefly, a 96 well plate was incubated with 14 ng/ml of rabbit HSA antibody in coating buffer at 4°C for 6 hours. Following incubation, the plate was washed and incubated overnight with PBS-Tween containing 5% milk. The following day the plate was washed again and a HSA standard curve (1.5-1000 μg/ml) and samples were added and incubated at 4C for 2 hours. Following incubation, the plate was washed and a rabbit anti-HSA antibody conjugated to HRP was added at a concentration of 130 ng/ml for 2 hours before a final wash. The plate was developed with the HRP substrate system (TMB), the reaction stopped with 1 M H_2_SO_4 _and optical density read at 450 nm. The limit of detection for this protocol was 0.3 ng/ml.

### Oxidation and derivatisation of the HSA and human tissue

A stock solution of 30 mg/ml of HSA was oxidised with equal volumes of 9% hydrogen peroxide and incubated at room temperature for 30 mins. 100 μl of the oxidised HSA was then derivatised with 100 μl of 10 mM DNPH in trifluroacetic acid and 100 μl of H_2_O. Samples were then incubated at room temperature for 45 mins, with vortexing every 10-15 mins. Derivatised protein was then precipitated on ice with 10% trichloroacetic acid for 30 mins. Following which the sample was centrifuged at 15,000 g for 5 mins and the supernatant removed. The pellet was then washed 3 times with 100 μl of ethanol/ethyl acetate (1:1) and then allowed to dry. Finally the pellet was broken up with sonication and re-suspended in 0.5 mls of 6 M guanidine hydrochloride in 0.5 M potassium phosphate (pH 2.5). The A_375 _was then measured and the carbonyl content of the oxidised HSA standard was then determined using ε_375 _22,000M^-1 ^cm^-1 ^(8). For baseline human tissue all samples were derivatised using the method described above.

### Carbonylated human serum albumin ELISA

To measure total levels of oxidised human serum albumin we adapted a previously published method used to measure total carbonylated protein (13). Briefly, a 96 well plate was incubated with 10 ng/ml of mouse anti-HSA antibody in coating buffer at 4°C for 6 hours. Following incubation, the plate was washed and incubated overnight with 0.1% PBS-Tween containing 5% soya milk. Following the overnight block, plates were washed and a derivatised HSA standard curve (0.04 - 45.4 μg/ml) and derivatised samples added and incubated at 4°C for 2 hours. Following the incubation with samples, the plate was washed and incubated with 1:5000 rabbit anti-dinitrophenyl (DNP) antibody, which had a specific antibody concentration of 1.0 - 1.7 μg/μl, for 2 hours at 4°C. Finally after washing, the plate was coated with 60 ng/ml of anti-rabbit HRP conjugate for 2 hours at 4°C. The plate was developed with TMB, the reaction stopped with 1 M H_2_SO_4 _and optical density read at 450 nm. The limit of detection for this was 0.02 ng/ml.

### Immunoblot

Samples were separated by electrophoresis on 10% SDS-polyacrylamide electrophoresis gels. The proteins were transferred to a nitrocellulose membrane (Bio-Rad) and blocked overnight with 20% milk. Blots were incubated with 1:1000 peroxidase conjugated anti-human albumin antibody (DAKO, Denmark) or 1:1000 anti-DNP antibody (Sigma, UK). Sites of antibody binding were visualised by Super signal west (Pierce, UK).

### Bicinchonic acid (BCA) assay

Total protein levels of lung homogenates were measured using a commercially available BCA assay from BioRad using a Human Serum Albumin (HSA) standard curve. Limit of detection for HSA was 4 μg/ml.

### Lactate dehydrogenase assay

LDH levels were measured in lung supernatant using a commercially available assay and LDH standard (0.9 - 2000 pg/ml) from Roche (Indianapolis IN, USA). To standardize for the maximum concentration of LDH present tissue was homogenised on ice using a sonicator set at amplitude of 2 microns; for 12 cycles of 10 seconds sonication followed by 20 seconds rest. Following sonication samples were centrifuged at 15,000 g for 15 minutes at 4°C, and supernatant removed for storage. The limit of detection of the assay was 0.5 pg/ml.

### Statistical analysis

Statistical analyses of results were carried out using Statview software™. The non-parametric Kruskal Wallis test was used to analyse all of the data except for the paired data where Non-parametric Wilcoxon Signed Rank analysis was carried out. P < 0.05 was considered as significant.

Multivariate linear regressions for COPD and non-COPD were performed to test for associations with HSA and carbonylated HSA. Confounding factors included for analyses of age, gender, COPD defined as (FEV_1_/FVC < 70%; FEV_1 _≤ 80% predicted) and smoking status using Statistica software™. COPD by smoking interactions were tested in the study by adding a multiplicative term to the regression models.

## Results

### Relationship between baseline levels of human serum albumin and GOLD I & II

**Figure 1:**
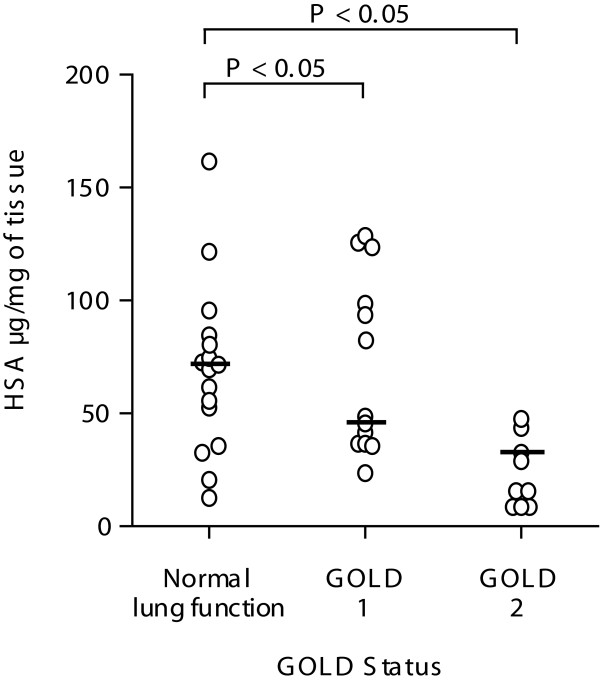
**Relationship between GOLD I and II patients and baseline levels of HSA**. Human lung tissue from 38 individuals classified using the GOLD guidelines was homogenised and adjusted for total protein. HSA levels were measured in lung homogenates using ELISA. The median is marked as a solid bar and expressed as μg/ml. Data was analysed using the non-parametric Kruskal Wallis test, P < 0.05 was considered to be statistically significant.

Parenchymal lung tissue from 38 individuals categorised as GOLD I (mild), II (moderate) or patients with no evidence of airway obstruction, was homogenised and the levels of HSA analysed using ELISA. As Figure 1 indicates, the level of HSA was decreased in lung tissue from GOLD II (median = 38.2 μg/ml, IQR = 15.5-48.9, P < 0.05) and GOLD I patients (median = 48.4 μg/ml, IQR = 36.6-93.4, P < 0.05) compared to individuals with normal lung function (median = 71.9 μg/ml, IQR = 52.2-87.6).

### Relationship between GOLD I & II and levels of carbonylated HSA

**Figure 2:**
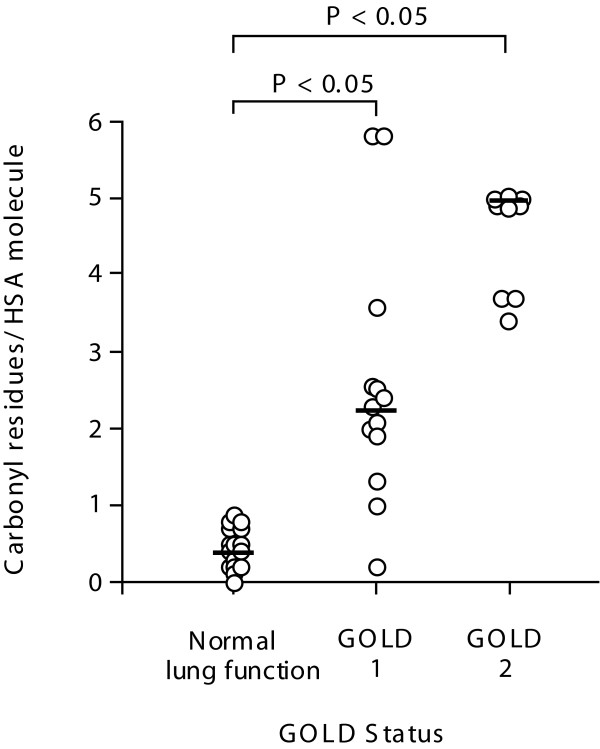
**Relationship between GOLD I and II patients and baseline levels of carbonylated HSA**. Human lung tissue from 38 individuals classified using the GOLD guidelines was homogenised, derivatised and the number of carbonyl residues measured using ELISA. The median is marked as a solid bar and expressed as carbonyl residues/HSA molecule. Data was analysed using the non-parametric Kruskal Wallis test, P < 0.05 was considered to be statistically significant.

The tissue homogenates shown in Figure 1 were also derivatised and the level of carbonyl residues per HSA molecule measured by ELISA. The numbers of carbonyl residues together with the values for total HSA shown in Figure 1 were used to calculate the number of carbonyl residues per HSA molecule. As shown if Figure 2 lung tissue from patients with normal lung function had very little carbonylated HSA (median = 0.40 carbonyl residues/HSA molecule, IQR = 0.2-0.7, P < 0.05). However we found the number of carbonyl residues per molecule of HSA was elevated in lung tissue from GOLD I patients (median of 2.3 carbonyl residues/HSA molecule, IQR = 1.9-2.5, P < 0.05) and was further elevated to a median of 5.0 carbonyl residues/HSA molecule in lung tissue from GOLD II patients (IQR = 4.0-7.6, P < 0.05).

### Re-classification of subjects using LLN for FEV_1_/FVC to define COPD

**Figure 3:**
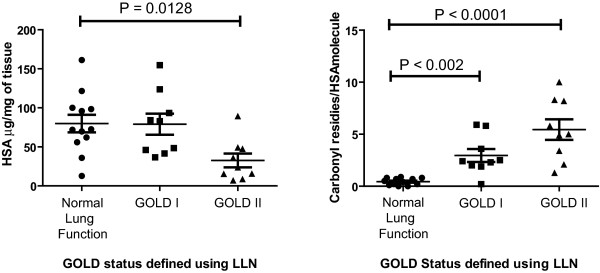
**Reclassification of subjects using LLN FEV_1_/FVC to define COPD**. Subjects from Figure 1 and 2 were re-classified using the lower limit of normal (LLN) for FEV_1_/FVC using prediction equations from the NHANES III and NSE studies to confirm COPD and then categorized by the GOLD guidelines. Data was analysed using the non-parametric Kruskal Wallis test, P < 0.05 was considered to be statistically significant.

The GOLD guidelines define airway obstruction as a fixed FEV_1_/FVC ratio of 0.70 which has been demonstrated to misdiagnose airway obstruction because FEV_1_/FVC varies with age, height and gender. Thus we re-classified the subjects in our study using the spirometry reference prediction equations from the NHANES III (16) and NSE (17) studies to confirm that the subjects defined with COPD by the GOLD guidelines did have a spirometry FEV_1_/FVC lower that the lower limit of normal FEV_1_/FVC (Table 2). From the 38 patients in this study, data on age, height and weight was only available for 31 of the subjects. All of the patients classified with COPD using the GOLD guidelines were also found to have obstructive lung disease using LLN FEV_1_/FVC. Using the LLN re-classified subjects we found individuals defined by the GOLD guidelines as GOLD II had significantly decreased levels of HSA compared to individuals with normal lung function and GOLD I patients (P = 0.0128, Figure 3A). We also observed that the number of carbonyl residues/HSA molecule was increased in individuals defined with COPD using the LLN for FEV_1_/FVC and GOLD guidelines stratification (Figure 3B).

### Relationship between baseline levels of human serum albumin and smoking status

**Figure 4:**
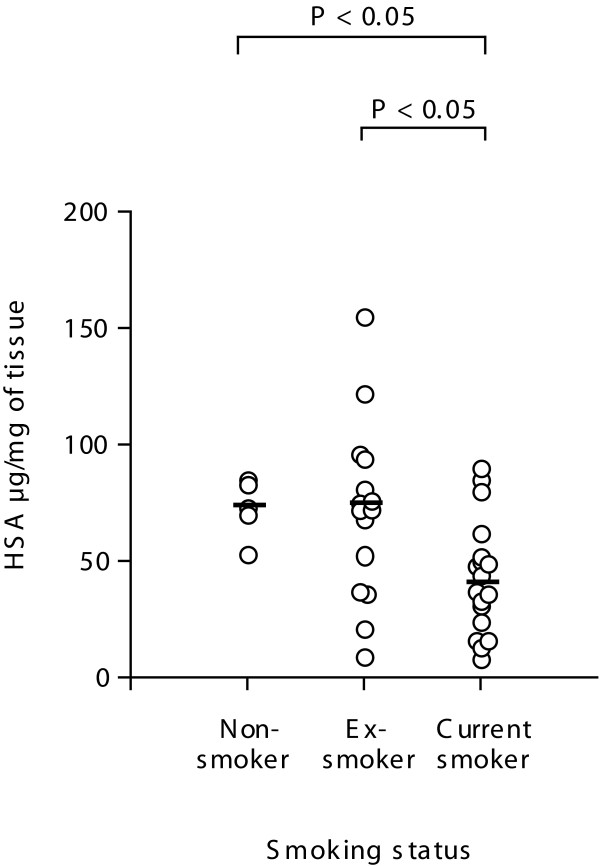
**Relationship between smoking status and baseline levels of HSA**. Human lung tissue from current smokers (*n *= 18), ex smokers (*n *= 15) and non-smokers (*n *= 5) was homogenised and adjusted for total protein. HSA levels were measured in lung homogenates using ELISA. The median is marked as a solid bar and expressed as μg/ml. Data was analysed using the non-parametric Kruskal Wallis test, P < 0.05 was considered to be statistically significant.

Having observed an inverse relationship between GOLD I and II patients and levels of HSA we turned our attention to the other clinical parameters collected in the study. When analysing smoking histories the data indicated that current smokers had lower levels of HSA (median = 43.3 μg/ml, IQR = 23.8-62.0, P < 0.05) compared to ex smokers (median = 71.9 μg/ml, IQR = 38.8-122.7) and non-smokers (median = 71.2 μg/ml, IQR = 44.9-80.3.7), as shown in Figure 4. We analyzed both COPD and smoking for an association with the levels of HSA in the study cohort. The data in Table 3 suggested an association with COPD and HSA levels (P = 0.001), and a significant interaction of COPD with smoking (P < 0.001).

**Table 3:** Analysis of COPD and smoking interactions on HSA and carbonylated HSA

HSA			
Term	Β	SE	P value

COPD	-0.6090	0.0029	0.001
Smoking status	-0.0651	0.0580	0.037
COPD × smoking	-0.3716	0.0170	<0.001

**Carbonylated HSA molecules/HSA molecule**			

Term	Β	SE	P value

COPD	-0.579	0.0053	0.001
Smoking status	-0.861	0.0035	0.001
COPD × smoking	-0.553	0.0033	0.007

Values are means ± plusorminus SD for non-continuous data unless otherwise stated

HSA, human serum albumin; COPD, ratio of air forcibly exhaled in one second (FEV1) to the forced vital capacity (FVC) pre-bronchodilator use (FEV_1_/FVC < 70%) and FEV1 ≤ 80% predicted; Smoking, current smoking history.

### Relationship between smoking status and levels of carbonylated HSA

**Figure 5:**
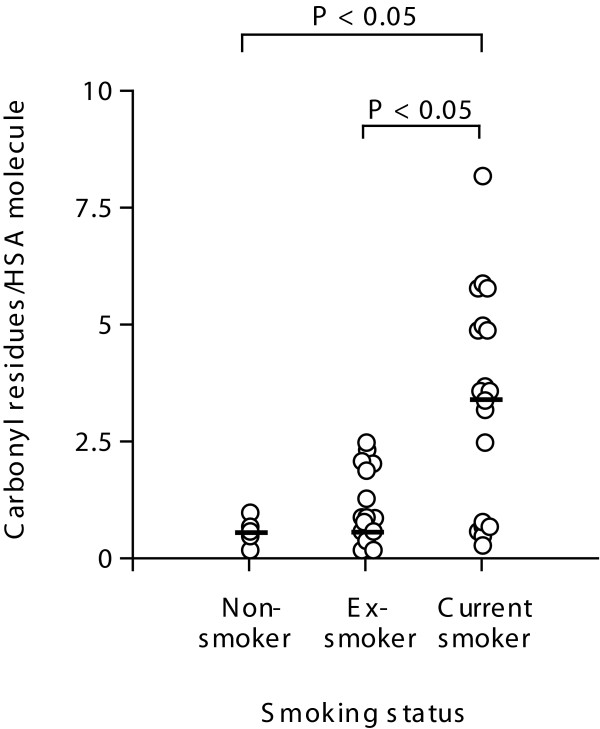
**Relationship between levels of carbonylated HSA and smoking status**. Human lung tissue from current smokers (*n *= 18), ex smokers (*n *= 15) and non-smokers (*n *= 5) was homogenised. Samples were derivatised and the number of carbonyl residues measured using ELISA. The median is marked as a solid bar and expressed as carbonyl residues/HSA molecule. Data was analysed using the non-parametric Kruskal Wallis test, P < 0.05 was considered to be statistically significant.

Since smoking status influenced baseline levels of HSA we next investigated whether levels of carbonylated HSA were also affected. We found no difference between the number of carbonylated HSA molecules in ex-smokers (median = 1.9 carbonyl residues/HSA molecule, IQR = 0.3-2.2) and the non-smokers (median = 1.51 carbonyl residues/HSA molecule, IQR = 0.6-2.2, Figure 5). This was in contrast to lung tissue from current smokers which exhibited a significantly greater number of carbonyl residues per HSA molecule (median = 3.60 carbonyl residues/HSA molecule, IQR = 0.7-4.9, P < 0.05).

We analyzed both COPD and smoking for an association with the levels of carbonylated HSA in the study cohort. The data in Table 3 suggested there was an association with COPD and smoking with carbonylated HSA levels (P = 0.001), and a significant interaction of COPD with smoking (P = 0.007).

### Degradation of HSA in human lung tissue

**Figure 6:**
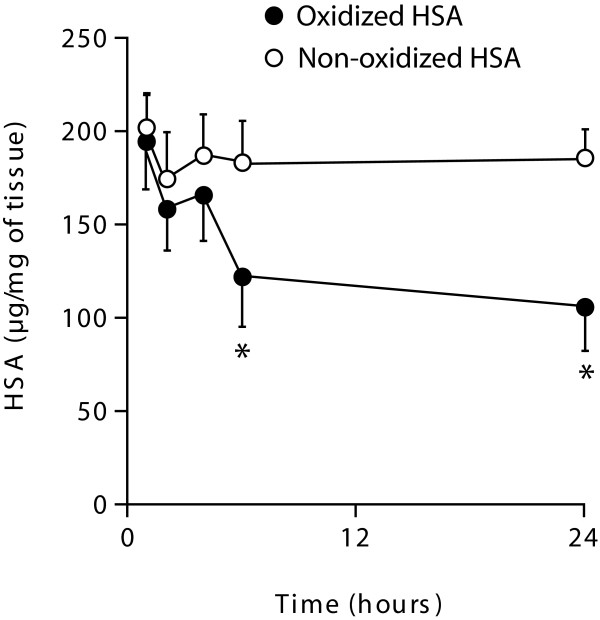
**Degradation of HSA and oxidised HSA in human lung tissue**. Human lung tissue (*n *= 12) was incubated with 200 μg/ml HSA (open circles) or 200 μg/ml oxidised HSA (filled circles) for 1, 2, 4, 6, and 24 hours. Samples were analysed for the levels of HSA using ELISA. Values given are the mean ± SEM and are expressed as μg/ml. The data was statistically analysed using the Wilcoxon-Signed rank test, * indicates a P value < 0.05.

**Figure 7:**
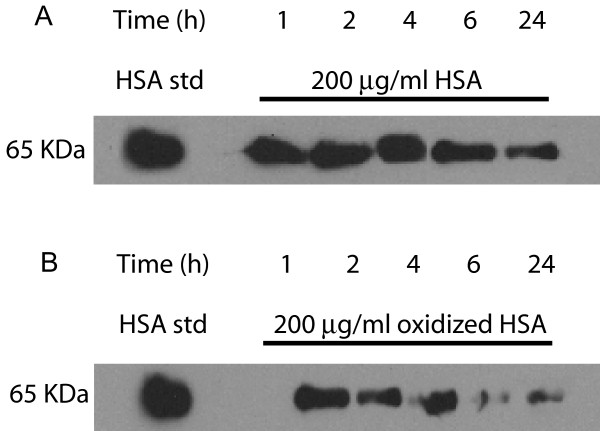
**Western blot analysis of HSA and oxidised HSA degradation in human lung tissue**. Human lung tissue (*n *= 12) was cultured with 200 μg/ml HSA or oxidised HSA and incubated for 1, 2, 4, 6, or 24 hours. Supernatants were separated on a 12% SDS-polyacrylamide gel and analysed for HSA expression using immunoblot. The supernatants cultured with HSA are depicted in Figure 7a and the supernatants cultured with oxidised HSA are shown in figure 7b. The blot depicted is a typical example of the molecular profile of HSA observed for all individuals in the study.

As we observed a reduction in the total levels of HSA in lung tissue from COPD patients and smokers (Figure 1, 3 and 4), but an increase in the number of carbonyl residues per molecule of HSA (Figure 2, 3 and 4), this indicated that oxidation may be effecting HSA turn over. Thus we investigated whether exogenously added oxidised HSA compared to unmodified HSA, is degraded in human lung tissue. To evaluate HSA degradation, human lung tissue explants from 12 individuals (6 ex, 5 current and 1 non-smoker, 5F/7 M, average FEV_1_/FVC = 0.64, average age = 68.1) were cultured with either 200 μg/ml HSA or oxidised HSA for 1, 2, 4, 6 and 24 hours and supernatants analysed using a HSA ELISA. As shown in Figure 6 when the tissue was incubated with non-oxidized HSA, the levels of HSA in the supernatant remained relatively constant over the 24 hour duration. In contrast, when tissue was incubated with oxidised HSA we observed a dramatic decrease in the detectable levels of HSA after 4 hours. Indeed, after 24 hours the levels of oxidised HSA had decreased to 105.7 μg/ml compared to 213.5 μg/ml for unmodified HSA, P < 0.05. The representative blot in Figure 7 for HSA in lung explant supernatants demonstrates the same pattern of rapid (A) oxidized HSA turnover over 24 hours compared to (B) unmodified HSA.

## Discussion

In the present study, we investigated the oxidation and degradation of HSA, an abundant sacrificial anti-oxidant, in explants of human lung tissue obtained from patients with and without COPD. We found parenchymal tissue from COPD patients who were current smokers contained lower levels of total HSA, but had proportionally greater levels of carbonylated HSA, compared to patients with normal lung function. Lung tissue from current smokers was also found to contain lower levels of HSA which was highly carbonylated compared to lung tissue from ex smokers and non-smokers. Cigarette smoking has been associated for many years with decreased levels of the anti-oxidants such as ascorbate and vitamin C (19-21). In addition, recent studies have shown decreased levels of ascorbic acid and Vitamin E in COPD patients during exacerbations compared to stable periods (22). However, this is the first study to provide evidence of reduced levels of the anti-oxidant HSA within parenchymal tissue from current smokers with COPD.

Serum albumin is one of the major antioxidants in the respiratory tract lining fluid, which also includes mucin, superoxide dismutase, glutathione, uric acid and ascorbic acid. The pathogenesis of COPD is thought to involve an increased oxidant burden both directly as a result of smoking and indirectly by the release of ROS which may not be adequately counterbalanced by the pulmonary antioxidant systems, resulting in net oxidative stress. Decreased levels of HSA in current smokers with COPD could therefore contribute to the excessive accumulation of oxidants which would lead to enhanced expression of pro-inflammatory mediators, inactivation of anti-proteinases and ultimately oxidative tissue injury. It is unlikely that current smokers with COPD are genetically predisposed to produce lower levels of HSA. Although single nucleotide polymorphisms in the gene have been documented, those that affect synthesis of the protein are extremely rare (23, 24). Alternatively it is possible that HSA like many genes emerging from the literature could be epigenetically regulated.

In an attempt to elucidate other possible mechanisms that could underpin the reduced expression of this anti-oxidant, we examined whether COPD and smoking affected the levels of oxidised HSA, and as a result its degradation. Our data demonstrate that the number of carbonyl residues per HSA molecule is increased in COPD patients. However within the study we were not able to obtain lung tissue from GOLD III and IV stage COPD patients to determine if the expression of HSA decreases with disease severity. However we could confirm that the subjects classified with COPD had obstructive lung function whether they were defined using the GOLD guidelines or the lower limit of normal for FEV_1_/FVC ratio using the prediction equation from the NHANES III (16) and NSE(17) studies. With both classifications we consistently found that GOLD II patients had decreased levels of HSA molecules which had a greater number of carbonylated residues. We also observed that lung explants from current smokers had elevated numbers of carbonyl residues per HSA molecule compared to those from ex and non-smokers. The association of COPD and smoking with levels of carbonylated HSA and a COPD × smoking interaction with levels of HSA indicates that the two cofactors are required to be present for the effects to manifest. In support of this, cigarette smoke has been shown to modify human plasma proteins, producing carbonyl proteins with lost sulfhydryl groups (25, 26). In the clinical setting it has been shown that the content of oxidised proteins recovered in BAL is greater in smokers compared with non-smoking control subjects (27). More importantly Rahman *et al *reported that plasma anti-oxidant activity is decreased acutely in cigarette smokers, following acute exacerbations in COPD patients (28). In addition oxidised HSA has previously been reported in BAL from COPD patients (29). As the parenchymal lung explants could not be inflated for histology, it was not possible to determine the localisation of HSA, which is a limitation of our study. The carbonylated HSA measured with the lung tissue could therefore be present in the intravascular space, extracellular fluid or intracellular environment. In the clinical setting it would thus be important to determine if the levels of carbonylated HSA were derived primarily from the lung or the systemic circulation. Ultimately independent of the source of HSA, decreased levels of the protein, could contribute to the oxidative burden within the lungs of smokers with COPD and potentially result in lung tissue damage.

Of particular note is our observation that lung tissue from ex smokers, defined as having given up smoking for at least 3 years, had the same mean concentration of carbonylated HSA as non-smokers. This may suggest that smoking cessation could prevent the elevated oxidation and degradation of HSA at least in part, contributing to the restoration of the oxidant/anti-oxidant balance within the lung. It is well documented that smoking cessation in addition to other therapies such as inhaled steroids and bronchodilators can be effective treatments for COPD, decreasing the accelerated decline in lung function and disease progression. If as our data suggests that the oxidant/anti-oxidant imbalance is resolved with smoking cessation it further supports the role of antioxidant disturbances in the progression of COPD. The data however can not indicate the time scale required for the resolution of smoking related oxidative stress within the lung.

In this current study we found that the proportion of carbonylated HSA was greatest in smokers with COPD. As carbonylated proteins are degraded more rapidly we hypothesised that in these patients' total levels of HSA are decreased due to rapid degradation of the carbonylated protein. Using an *in vitro *lung tissue culture system we added exogenous oxidised HSA to model the effects of oxidised HSA within the extracellular fluid of the lung. In support of this hypothesis our *in vitro *data demonstrated that oxidised HSA was degraded more rapidly than unmodified HSA in cultured human lung tissue explants, when analysed by ELISA and western blot. Larger molecular proteins such as albumin are primarily cleared from the lung by paracellular mechanisms, into the systemic circulation. However, as the supernatant and tissue were analysed in our model it suggests that carbonylated HSA could be degraded by the parenchymal lung explants. In support of this finding, it has been demonstrated that both albumin and other high molecular weight proteins can be directly cleared by the epithelium through epithelial receptor mediated endocytosis or pinocytosis, and these proteins are catabolised through lysosomal degradation (30-32). Recent evidence suggests that oxidation of HSA decreases its denaturation enthalpy, suggesting that oxidation of HSA renders it to be denatured more easily (33). The precise mechanisms involved in the metabolic turnover of HSA have not been fully elucidated. They are thought also to involve the uptake of damaged proteins by type A scavenger receptors found on macrophages and the sinusoidal liver epithelial cells (34, 35). The tissue culture experiments were performed on parenchymal tissue from donors with and without COPD and different smoking histories. Although no differences were observed between the responses of parenchymal tissue from different donors, the sample size was too small for statistical analysis, which is a limitation to determine the effects of smoking and disease on HSA turnover.

In summary, our study provides further evidence for the role of oxidative stress in current smokers with COPD and is the first study to evaluate the effect of oxidation on HSA degradation in human lung tissue. HSA is currently used clinically to maintain colloid osmotic pressure and is also viewed as an important antioxidant in patients with damaged vascular endothelium and patients with acute lung injury (7, 36, 37). Our data suggests that it might also be important not only to consider oxidised HSA as a marker of oxidative stress in current smokers with COPD, but also the potential therapeutic role of HSA in the homeostasis of the oxidant/anti-oxidant balance, where there is a large unmet clinical need.

## Competing interests

The authors declare that they have no competing interests.

## Authors' contributions

TLH participated in the study design carried out the tissue culture studies, immunoassays, performed the statistical analysis and drafted the manuscript. MS, LZ, WT and TT participated in patient data collection, statistical analysis and manuscript revision. JAW conceived of the study, participated in its design, analysis and manuscript preparation. All authors read and approved the final manuscript.
